# CRISPR/Cas13d targeting GZMA in PARs pathway regulates the function of osteoclasts in chronic apical periodontitis

**DOI:** 10.1186/s11658-023-00477-2

**Published:** 2023-08-25

**Authors:** Tingting Jia, Fang Yuan, Jingqiao Tao, Gang Wang, Xianhua Zhang, Bin Zhang, Hongbo Li

**Affiliations:** 1 Department of Stomatology, The First Medical Centre, Chinese PLA General Hospital, Beijing, China; 2 Department of Oncology, The Fifth Medical Centre, Chinese PLA General Hospital, Beijing, China; 3 Department of Stomatology, Southern Medical Branch of PLA General Hospital, Beijing, China; 4 Medical School of Chinese PLA, Beijing, China

**Keywords:** GZMA, CRISPR/Cas13d, Osteoclast, Chronic apical periodontitis

## Abstract

Chronic apical periodontitis is a prevalent oral disease characterized by bone loss, and its underlying mechanisms remain unclear. This study aimed to investigate the role and mechanism of the serine protease GZMA in osteoclasts during chronic apical periodontitis. To address this, we employed crRNA/Cas13d to inhibit GZMA expression and examined its impact on osteoclast behavior. Our findings revealed that GZMA plays a significant role in promoting osteoclast cell proliferation while inhibiting cell apoptosis. Additionally, the inhibition of GZMA led to a notable increase in miR-25-3p expression, which, in turn, downregulated the expression of TGF-β. Consequently, the reduction in TGF-β expression led to a decrease in PAR1 expression within the PARs pathway. These results suggest that GZMA might serve as a promising therapeutic target for the treatment of chronic apical periodontitis. Furthermore, our study highlights the potential of targeting GZMA using crRNA/Cas13d as a valuable approach for future therapeutic interventions.

## Introduction

Apical periodontitis (AP) is a global epidemic, which is characterized by microbial infection in the dental pulp causing inflammation and bone destruction in the periapical tissue [1]. Chronic apical periodontitis (chronic apical periodontitis, chronic AP) is mainly caused by long-term stimulation of pathogenic irritants in the root canal, which leads to tissue damage around the root apex through the apical foramen and root canal system, which is characterized by bone resorption around the root apex of the tooth. AP is the most common inflammatory disease associated with jaw teeth. Compared with other bones in the body, the jaw bone creates a direct path to the bone marrow due to the presence of teeth. When the pulp is necrotic and infected, the jaw bone lacks an epithelial barrier to resist infection and inflammatory factors. Therefore, the tissue and immune response are essential to prevent the spread of the infectious agent bone marrow. Microbial antigens from root canal infection can stimulate specific and non-specific immune responses in the periapical tissues [2]. Macrophages, mast cells, T cells and neutrophils, as well as various cytokines secreted by them [3], chemokines, RANK/RANKL/OPG (nuclear factor κB ligand/osteoprotectin receptor activator ). The system is closely involved in the formation of AP/ chronic AP. The interaction of a large number of inflammatory cells and stimuli can affect and change the state and progress of AP/ chronic AP disease [4]. It is crucial to note that chronic AP has a substantial prevalence and significant impact on oral health globally. Understanding its pathogenesis and the underlying mechanisms is vital for the development of effective treatment strategies.

Bone resorption is mainly caused by the imbalance in the formation and differentiation of osteoblasts and osteoclasts [5]. In chronic AP, under the stimulation of bacteria and toxins in the root canal, lymphocytes, hypertrophic cells, and macrophages are released and aggregated in the periapical tissue; mast cells can degranulate and produce a variety of inflammatory substances, such as vascular activity Mediator [6], macrophages can produce pro-inflammatory or anti-inflammatory substances, which act on the development or repair of chronic AP lesions by secreting soluble IL-1α, TNF-α, IL-6, TGF-β, etc. Mechanism studies believe that the cytokines secreted by these cells can promote the activation and differentiation of osteoclasts, the activation and proliferation of fibroblasts, the production of collagen and the formation of new blood vessels, etc., in the initiation and regulation of the chronic AP inflammation process [7].

Granzyme A (GZMA) is a serine protease, which is mainly found in the granzyme secreted by cytotoxic cells and has the most abundant content. Although the biological role of GZMA is uncertain, it seems to be partly through inducing monocytes and macrophages to produce pro-inflammatory cytokines, such as interleukin 1 (IL-1), IL-6, IL-8, and tumor necrosis factor (TNF)), enhance the inherent response of host inflammation and autoimmune diseases [8]. Adhesive monocytes are particularly affected by GZMA, indicating that granzyme may stimulate the immune microenvironment of these cells in the body and produce pathological results. Recently, it was discovered that GZMA -deficient mice showed protection from bacterial sepsis without controlling the action of the pathogen [9]. In addition, NK cells containing GZMA have been found to stimulate osteoclast production in joints, although the mechanism is still unclear [10]. In addition, Llipsy Santiago et al. [11] recently reported that GZMA -deficient mice showed reduced osteoclast production in vivo and reduced collagen-induced arthritis symptoms, manifested by serum levels of pro-inflammatory cytokines, joint damage, and affected joints. The level of bone erosion is alleviated, which indicates that the activity of osteoclasts is reduced in the absence of GZMA; at the in vitro cell level, bone marrow cells treated with GZMA can produce multinucleated cells that meet the criteria for mature osteoclasts. These findings indicate that GZMA is involved in the formation and activation of osteoclasts under inflammatory stimulation, and its role in chronic AP has not been reported yet.

Recently discovered RNA-targeting CRISPR/Cas13d system which contains a corresponding CRISPR RNA and a single Cas13d nuclease has shown its great potential in gene silencing without altering the whole genome [12]. In vivo, the CRISPR/Cas13d system demonstrated a more efficient RNA knockdown property when compared to traditional RNAi technology [13]. Besides, various studies demonstrate that CRISPR/Cas13d can be ideal modifiable RNA-targeting platforms for further transcriptome engineering to regulate gene expression [14].

In this study, we would like to investigate the role of GZMA in chronic AP. CRISPR/Cas13d was used to inhibit the expression of GZMA to investigate the function and mechanism of GZMA in chronic apical periodontitis. Our results demonstrated that the expression of GZMA was inhibited significantly by our engineered CRISPR/Cas13d system. The proliferation of osteoclasts was suppressed obviously after inhibition of GZMA. What’s more, GZMA inhibited the apoptosis of osteoclasts in chronic AP. Mechanically, GZMA promoted the ability of osteoclasts through suppressing exportin 5 (XPO5) and promoting miR-25-3p to regulate PARs pathway in chronic AP.

## Materials and methods

### Cell culture

RAW 264.7 cell line (RRID:CVCL_0493) was obtained as a model cell line from chronic apical periodontitis acts as a precursor for OC formation, responding to osteoclastogenic stimuli, including RANKL[15].

### Cell proliferation assay

The viability of cells was detected by CCK-8 assay. 2000 cells/well were seeded in 96-well plates. 10 µl CCK-8 reagent was added to each well for 30 minutes. A microplate reader (Thermo Labsystems, Vantaa, Finland) was used to measure the absorbance at 450 nm.

### RNA extraction and quantitative real time-PCR (qRT-PCR)

TRIzol reagent was used to isolate total RNA from cells according to the manufacturer’s instructions. The complementary DNA (cDNA) was synthesized from total RNA using a PrimeScript RT Reagent Kit with gDNA Eraser (Takara., Dalian, China) or a commerical miRNA reverse transcription PCR kit (RiboBio, Guangzhou, China). The SYBR Premix Ex TaqTM kit (Takara, Dalian, China) was used for the quantitative real-time PCR (qRT-PCR) analysis on the Roche lightcycler 480 Real-Time PCR System. Levels of GAPDH or U6 were used as an endogenous control to normalize the differences of mRNA and miRNA. All assays were carried out at least three times.

### Cell apoptosis assays

Cell apoptosis was determined by caspase 3 ELISA assay and flow cytometry assay. After incubation for 48 h, the caspase 3 ELISA assay kit was used to detect the activity of apoptosis marker caspase 3. A microplate reader was used to measure the OD values. Besides, GZMA ELISA Kit (cat. no. BMS2232, Invitrogen, USA), TGFB ELISA Kit (cat. no. BMS2065, Invitrogen, USA), PAR1 ELISA kit (cat. no. EH358RB, Invitrogen, USA) and exportin 5 ELISA Kit (cat. no. abx151460, Abbexa, UK) were used for ELISA according to the manufacturer's protocol. In order to understand the accurate rate of apoptotic cells, we used the flow cytometry assay. After transfection for 48 h, cells were collected and then washed by PBS. Besides, cells were mixed with Annexin V-FITC (AV) and Propidium Iodide (PI), and incubated in darkness for 15 min. The rate of apoptotic cells was measured by a flow cytometer. Each experiment was repeated at least three times.

### Dual luciferase reporter assay

Cells (1 × 10^5^ per well) were cultured in 24-well plates and transfected with control or experimental groups. After 48 h transfection, the luciferase activity was measured using the dual luciferase assay system (Promega, Madison, WI, USA) according to the manufacturer’s protocol. The experiments were performed at least three times.

### Statistical analysis

Each experiment was performed in triplicate. All quantitative data were expressed as mean ± standard deviation (SD). Statistical analyses were performed by using SPSS 22.0 software (IBM). Statistical significance was tested by Student’s t-test, Chi square test or ANOVA. P < 0.05 was considered to be statistically significant.

## Results

### The GZMA expression was suppressed by CRISPR/Cas13d

**Fig. 1:**
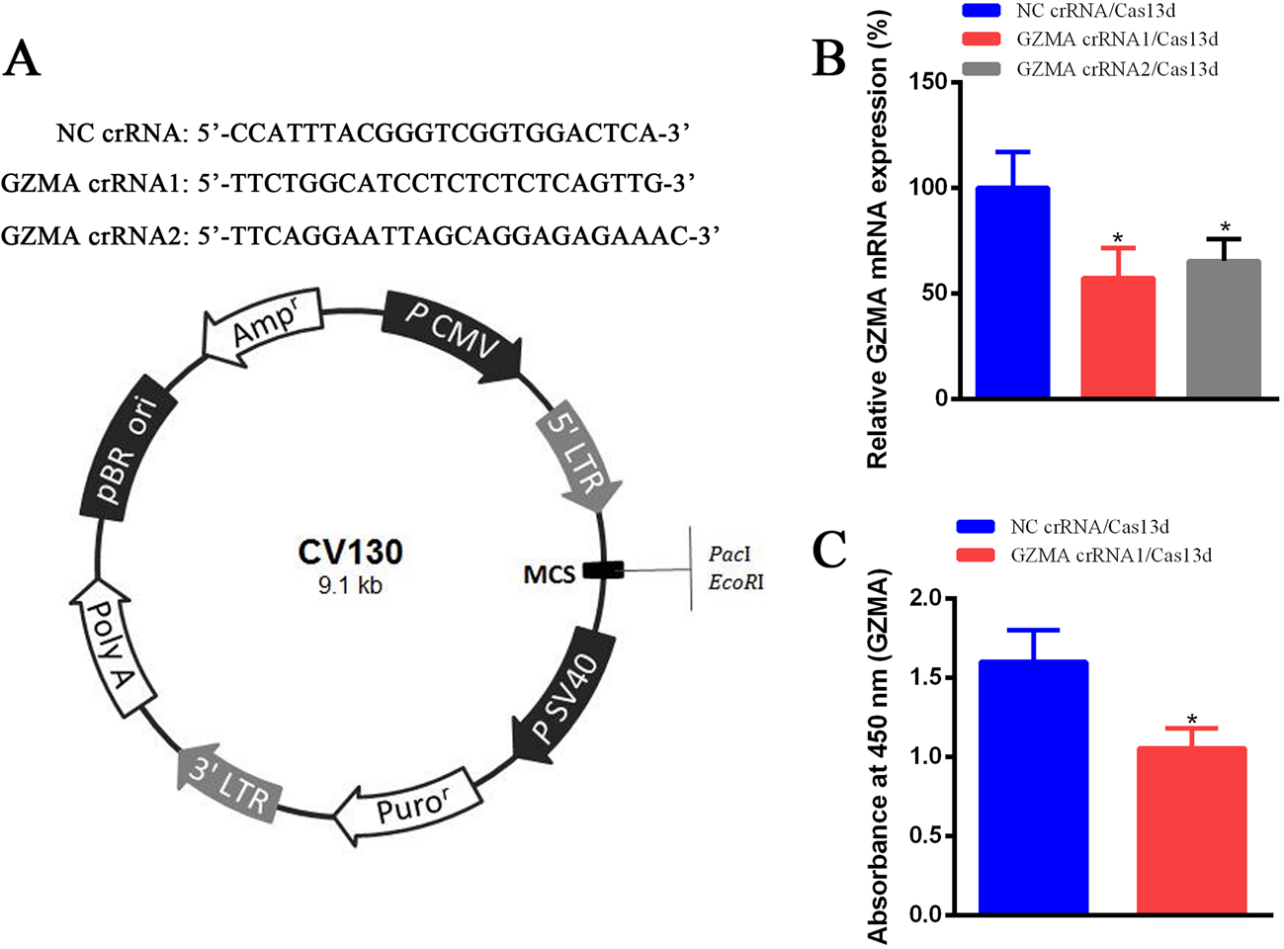
The expression of GZMA was inhibited significantly by crRNA/Cas13d. **A** The plasmid profile and sequences of crRNA were presented. **B** The GZMA mRNA expression was suppressed dramatically by GZMA crRNA/Cas13d. **C** The GZMA protein expression was restrained via GZMA crRNA1/Cas13d using ELASA assay. *Means *P* < 0.05

According to the design principle of crRNA, we constructed two crRNA targeting GZMA based on the vector CV130 (Fig. 1A). Sequences of crRNA targeting GZMA and negative control were also shown in Fig. 1A and inserted into CV130 at the restriction enzyme cutting sites between PacI and EcoRI. Results demonstrated that both GZMA crRNA1 and crRNA2 guide Cas13d digests GZMA and inhibit the mRNA expression of GZMA significantly (Fig. 1B). And we choose GZMA crRNA1 for further study. The result of ELISA indicated that the GZMA protein level was also suppressed markedly using Cas13d with the guidance of GZMA crRNA1 (Fig. 1C).

### GZMA promoted the proliferation and inhibited the apoptosis of osteoclast in chronic apical periodontitis

**Fig. 2:**
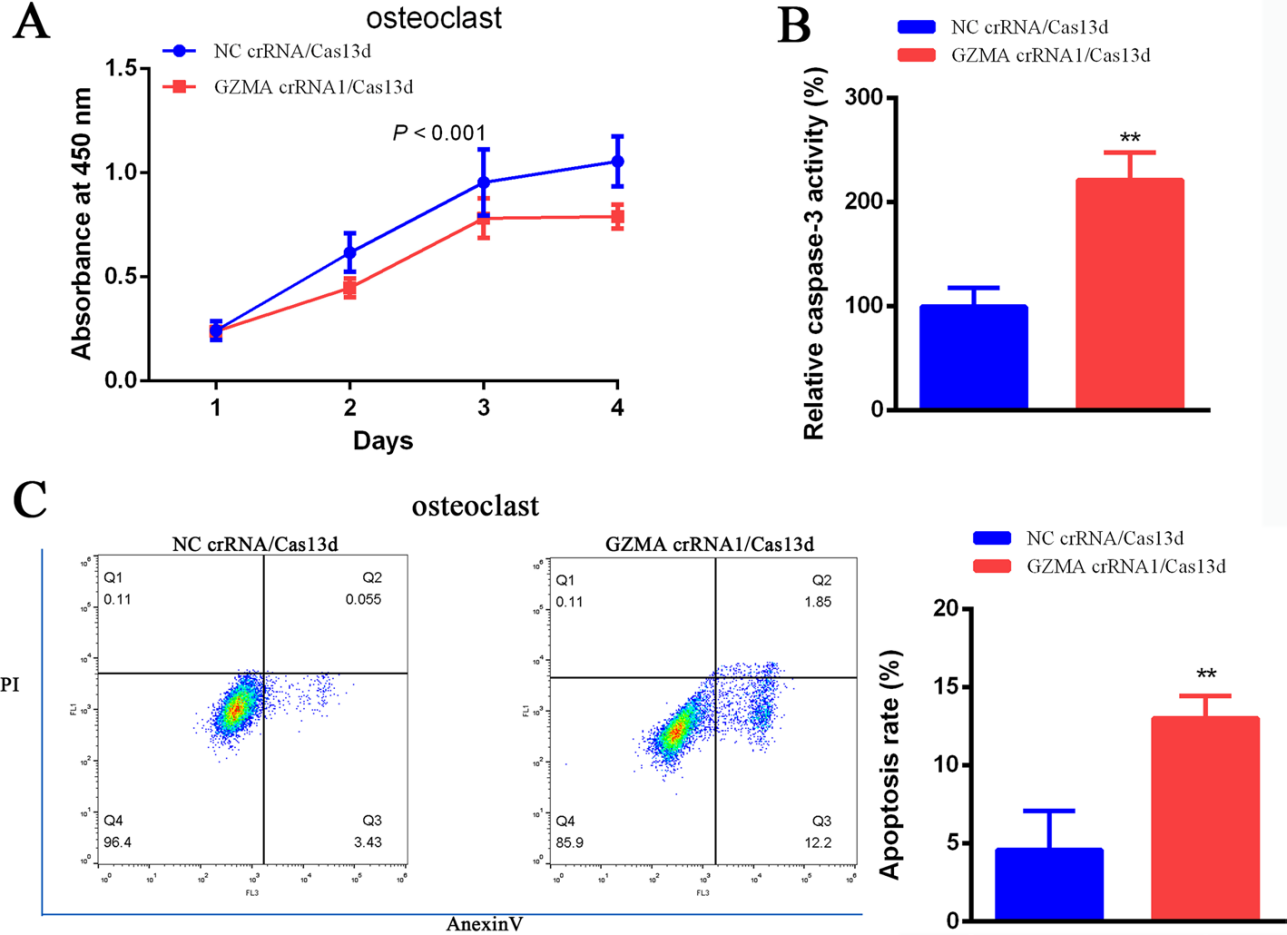
GZMA promoted the cell proliferation of osteoclast and inhibited cell apoptosis markedly in chronic apical periodontitis. **A** Osteoclast cell growth was suppressed significantly after inhibition of GZMA via crRNA/Cas13d. **B** Caspase-3 activity was increased obviously after suppression of GZMA via crRNA/Cas13d using ELISA assay. **C** Apoptosis rate was promoted dramatically after decrease of GZMA via crRNA/Cas13d using flow cytometry. *Means *P* < 0.05, ** means *P* < 0.01

In order to investigate the function of GZMA in chronic apical periodontitis, CCK-8 assay, ELISA and flow cytometry were used to detect the growth and apoptosis of osteoclast. The proliferation of osteoclast was inhibited significantly with the transfection of GZMA crRNA/Cas13d compared with the negative control (Fig. 2A) using CCK-8 assay. However, the activity of apoptosis marker caspase-3 was increased obviously in GZMA crRNA/Cas13d group compared with the negative control (Fig. 2B). Besides, flow cytometry experimental method was also used to study the effect of GZMA on apoptosis again. As shown in Fig. 2C, four quadrants were represented and right lower quadrant was on behalf of the rate of early apoptotic cells. The rate of early apoptotic cells was promoted dramatically after suppression of GZMA using GZMA crRNA1/Cas13d in comparison with NC crRNA1/Cas13d. In short, the growth of osteoclast was promoted by GZMA, while the apoptosis was restrained.

### CRISPR/Cas13d regulated the function of GZMA through controlling the expression of PAR1 in PARs pathway in chronic apical periodontitis

**Fig. 3:**
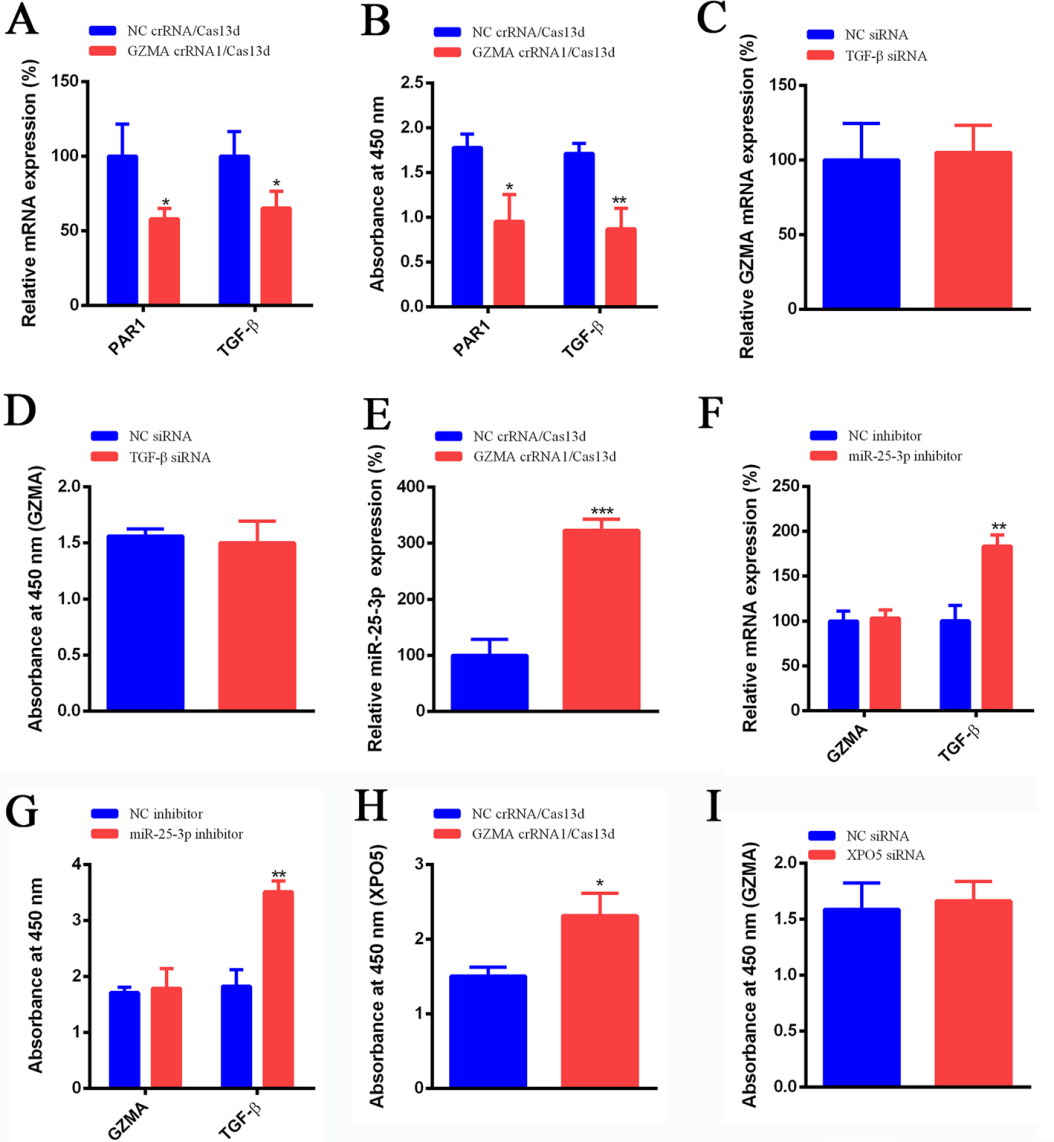
CRISPR/Cas13d regulated the function of GZMA through controlling the expression of miR-25-3p and PAR1 in PARs pathway in chronic apical periodontitis. **A**, **B** The PAR1, TGF-β mRNA and protein expression levels were decreased significantly after inhibition of GZMA via crRNA/Cas13d. **C**, **D** The GZMA mRNA and protein expression levels were no changed after knockdown of TGF-β. **E** The miR-25-3p expression was promoted significantly after inhibition of GZMA. **F**, **G** After transfected with miR-25-3p inhibitor, the GZMA mRNA and protein expression levels were no changed. While, the TGF-β mRNA and protein expression levels were increased dramatically. **H** The expression of XPO5 was increased significantly. **I** The GZMA protein expression level was no changed after transfection of XPO5 siRNA. *Means *P* < 0.05, **means *P* < 0.01, ***means *P* < 0.001

One recent study reported that GZMK activates protease-activated receptor 1 (PAR1) enhancing activation of monocytes and wound healing in endothelial cells [16]. Wang et al. demonstrated that TGF-β induced the expression of PAR-1 involving in PARs pathway and promotes tumor progression, osteoclast differentiation in giant cell tumor of bone [17]. Inspired by these studies, we would like to test the effect of GZMA on PAR1 and TGF-β. Our results indicated that the mRNA of PAR1 and TGF-β was both inhibited significantly with the suppression of GZMA using CRISPR/Cas13d (Fig. 3A). Similarly, the protein expression levels of AR1 and TGF-β was also both suppressed dramatically in GZMA crRNA1/Cas13d group compared with the negative control (Fig. 3B). Inversely, there was no difference on the mRNA and protein expression of GZMA after the inhibition of TGF-β (Fig. 3C, D). It manifested that GZMA regulated the expression of PAR1 and TGF-β upstream. Genz et al. proved that the expression of TGF-β was inhibited significantly by overexpression of miR-25-3p [18]. In our study, the expression of miR-25-3p was increased dramatically when GZMA expression level was decreased by crRNA/Cas13d (Fig. 3E). There was no effect on GZMA expression after inhibition of miR-25-3p, while the mRNA and protein expression of TGF-β was promoted with the transfection of miR-25-3p inhibitor (Fig. 3F, G). Studies demonstrated that exportin 5 (XPO5) can transport precursor miRNAs (pre-miRNAs) from the nucleus to the cytoplasm [19]. Any alterations of XPO5, resulting from either epigenetic change, abnormal expression level or posttranslational modification, could affect the expression of miRNA including miR-25-3p and have profound effects on cell proliferation and apoptosis [20]. Our data indicated that the expression of exportin 5 was inhibited significantly after suppression of GZMA via crRNA/Cas13d (Fig. 3H). While the suppression of exportin 5 has no effects on the expression of GZMA in osteoclasts in chronic apical periodontitis.

### The phenotype of GZMA in osteoclasts was rescued by miR-25-3p and the schematic diagram of GZMA was presented

**Fig. 4:**
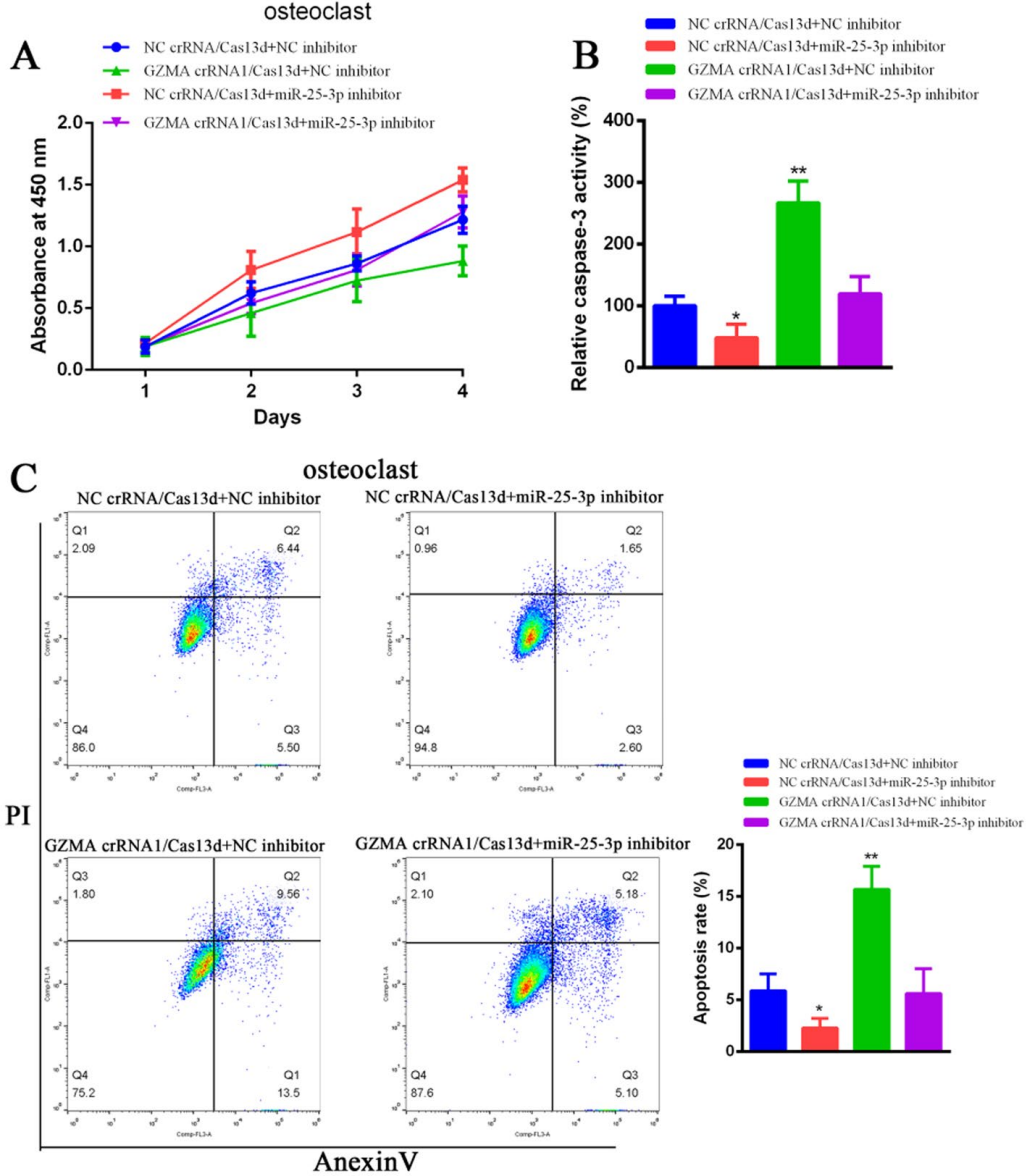
The phenotype of GZMA in osteoclasts was rescued by miR-25-3p. **A** The inhibitory effect of GZMA crRNA1/Cas13d on osteoclast cell proliferation was rescued obviously by miR-25-3p inhibitor. **B** The promotion of caspase-3 activities in GZMA crRNA1/Cas13d group was reversed dramatically by the suppression of miR-25-3p. **C** The increase of apoptosis rate was also reversed significantly by transfection of miR-25-3p inhibitor using flow cytometry assay. *Means *P* < 0.05, ** means *P* < 0.01

**Fig. 5:**
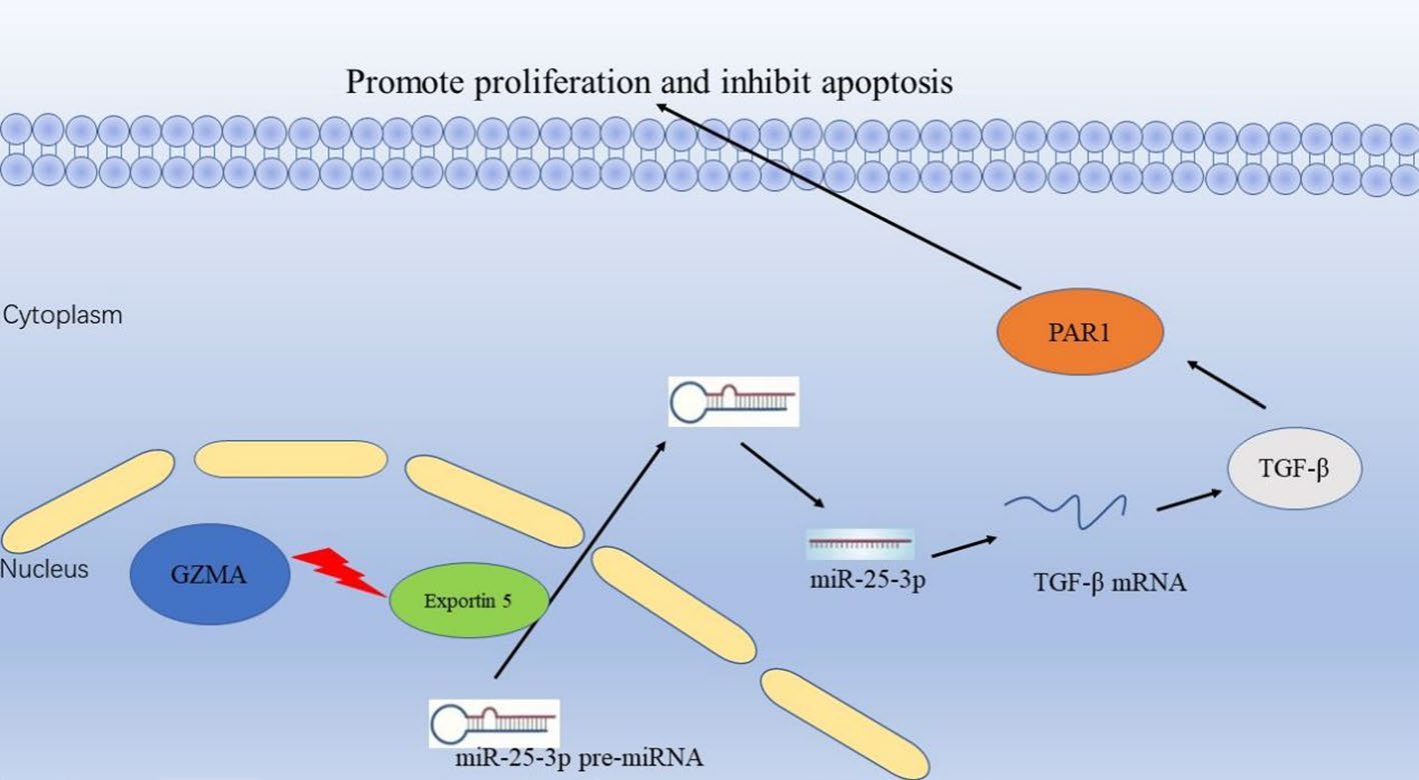
The schematic diagram of GZMA in osteoclasts. GZMA degrades exportin 5 that transports miR-25-3p pre-miRNA from the nucleus to the cytoplasm of osteoclast, and suppresses the expression of miR-25-3p. TGF-β promotes the expression of PAR1 and finally facilitates cell growth, inhibited cell apoptosis in osteoclasts

Our results as mentioned above indicated that GZMA promoted the growth, inhibited apoptosis of osteoclasts and suppressed the miR-25-3p expression. In order to verify that GZMA regulated miR-25-3p in osteoclasts, we used rescue assays to investigate the relationship between GZMA and miR-25-3p. As shown in Fig. 4A, the inhibitory effect of GZMA crRNA1/Cas13d on osteoclast cell proliferation was rescued obviously by miR-25-3p inhibitor. Furthermore, the promotion of caspase-3 activities in GZMA crRNA1/Cas13d group was reversed dramatically by the suppression of miR-25-3p (Fig. 4B). Besides, the increase of apoptosis rate was also reversed significantly by transfection of miR-25-3p inhibitor using flow cytometry assay (Fig. 4C). In summary, GZMA facilitated cell proliferation and suppressed cell apoptosis in osteoclasts via regulating the expression of miR-25-3p. GZMA degrades exportin 5 that transports miR-25-3p pre-miRNA from the nucleus to the cytoplasm of osteoclast, and suppresses the expression of miR-25-3p from miR-25-3p pre-miRNA. The mRNA and protein levels of TGF-β are increased significantly owing to weak inhibitory role of low expression of miR-25-3p. Finally, as mentioned in previous studies, TGF-β promotes the expression of PAR1 in PARs pathway in chronic apical periodontitis. The mechanism of GZMA in osteoclasts is represented in Fig. 5.

## Discussion

Granzyme A (GZMA) is one kind of serine proteases according to the classification of cleavage specificity [21]. GZMA is the most abundant serine protease in killer cell cytotoxic granules [22]. In colitis [23], endotoxicosis [24], rheumatoid and viral arthritis [11], GZMA was proved to act as pro-inflammatory mediators in the process of different inflammatory disorders. Studies demonstrated that GZMA deficiency may also be a predisposing factor for type 1 diabetes [25]. GZMA deficiency leads to abnormal accumulation of single strand DNA in dendritic cells and NK cells in islets and spleen. As an endogenous signal, single strand DNA can also stimulate the production of local IFN-I to aggravate the destruction of pancreatic islets [26]. The mechanism of GZMA in different diseases is different, and the expression level of GZMA in serum or tissue is different. The role of GZMA expression level in disease diagnosis and efficacy evaluation needs further study [22]. Another kind of serine proteases, GZMK, activates protease-activated receptor 1 (PAR1) that is main component in PARs pathway enhancing activation of monocytes and wound healing in endothelial cells [16]. Furthermore, Tudpor et al. reports that PAR1 increases bone mass and trabecular thickness and bone resorption at the bone remodeling procedure [27]. However, the role of GZMA in inflammation and bone remodeling process is still unknown. Chronic apical periodontitis means the chronic inflammation of periapical tissues owing to the long-term presence of infection and pathogenic irritants in the root canal, which is produced by the formation of inflammation and bone loss [28]. Till now, no studies have illustrated the function and mechanism of GZMA in the chronic apical periodontitis.

In order to suppress the expression of GZMA, the CRISPR/Cas13d system, a more efficient RNA knockdown tool [13], was used to target GZMA in this study. Studies also indicated that engineered CRISPR/Cas13d would be ideal modifiable RNA-targeting platforms to regulate gene expression for the therapy of disease [29, 30]. GZMA crRNA/Cas13d inhibited the expression of GZMA significantly in osteoclast in chronic apical periodontitis in this study. The results of proliferation and apoptosis assays presented that GZMA promoted cell proliferation and suppressed cell apoptosis dramatically in osteoclast.

Next, the mechanism of GZMA is also studied. To investigate the relationship between GZMA and PAR1, we used GZMA crRNA/Cas13d to inhibit the expression of GZMA and found that the expression of PAR1 in osteoclast was also suppressed obviously. A previous study suggests that TGF-β induced the expression of PAR-1 involving in PARs pathway and promotes osteoclast differentiation [17]. Then, we proved that GZMA also increased the expression of TGF-β. Lots of studies have demonstrate that miRNA regulated various biological processes in cells including controlling gene expression [31]. Thus, the correlation between miRNA and TGF-β was further studied. Genz et al. showed that the expression of TGF-β was inhibited significantly by overexpression of miR-25-3p [18]. We knock down the expression of GZMA and found that the expression of miR-25-3p was increased significantly unexpectedly. Exportin 5 was a critical component for the production of miRNA and loss-function of exportin 5 limits the mature of miRNA including miR-25-3p [20]. It showed that GZMA downregulated the expression of exportin 5 and restrain the expression of mature miR-25-3p. The result of rescue assay demonstrated that GZMA promoted osteoclast cell proliferation and inhibited cell apoptosis via regulating miR-25-3p to facilitate the PAR1 expression in PARs pathway. Additional investigations are required to comprehensively understand the signaling pathways and molecular interactions underlying GZMA-mediated regulation of PAR1 expression. This may involve exploring the involvement of other factors in this regulatory network.

In conclusion, the findings of this study shed light on the clinical implications and translational potential of targeting GZMA in the treatment of chronic apical periodontitis. The advantages of using the CRISPR/Cas13d system for therapeutic purposes, such as efficient RNA knockdown, make it an attractive approach. However, further research is required to overcome challenges and explore the full potential of this system in clinical applications for chronic apical periodontitis treatment. Other CRISPR systems with potential for disease treatment, such as CRISPR/Cas9 [32] and CRISPR/Cas12 [33], can also be considered for use.

## Data Availability

All data and materials used in this study are available upon resonable request from the corresponding author.

## Abbreviations

AP: Apical periodontitis
TNF: Tumor necrosis factor
IL: Interleukin
RANK/RANKL/OPG: Receptor activator of nuclear factor kappa-Β ligand/osteoprotegerin
TGF-β: Transforming growth factor beta
GZMA: Granzyme A
CRISPR/Cas13d: Clustered Regularly Interspaced Short Palindromic Repeats/Cas13d nuclease
RNAi: RNA interference
XPO5: Exportin-5
PARs: Protease-activated receptors
